# miRNAs in Serum Exosomes for Differential Diagnosis of Brain Metastases

**DOI:** 10.3390/cancers14143493

**Published:** 2022-07-18

**Authors:** Silvia Catelan, Debora Olioso, Alessandra Santangelo, Chiara Scapoli, Anna Tamanini, Giampietro Pinna, Francesco Sala, Giuseppe Lippi, Antonio Nicolato, Giulio Cabrini, Maria Cristina Dechecchi

**Affiliations:** 1 Section of Neurosurgery, Department of Neurosciences, Biomedicines and Movement, University of Verona, 37126 Verona, Italy; silvia.catelan28@gmail.com (S.C.); francesco.sala@univr.it (F.S.); 2 Section of Clinical Biochemistry, Department of Neurosciences, Biomedicines and Movement, University of Verona, 37126 Verona, Italy; debora.olioso@univr.it (D.O.); santangeloale84@gmail.com (A.S.); giuseppe.lippi@univr.it (G.L.); giulio.cabrini@unife.it (G.C.); 3 Department of Life Sciences and Biotechnology, University of Ferrara, 40121 Ferrara, Italy; scc@unife.it; 4 Section of Molecular Pathology, Department of Pathology and Diagnostics, University Hospital of Verona, 371234 Verona, Italy; anna.tamanini@aovr.veneto.it; 5 Institute of Neurosurgery A, Department of Neurosciences, University Hospital of Verona, 371234 Verona, Italy; giampietro.pinna@aovr.veneto.it; 6 Institute of Neurosurgery B, Department of Neurosciences, University Hospital of Verona, 371234 Verona, Italy; 7 Section of Stereotaxy, Department of Neurosciences, University Hospital of Verona, 371234 Verona, Italy; antonio.nicolato@aovr.veneto.it; 8 Center on Innovative Therapies for Cystic Fibrosis, Department of Life Sciences and Biotechnology, University of Ferrara, 40121 Ferrara, Italy

**Keywords:** brain metastases, gamma knife, exosomes, circulating miRNAs

## Abstract

**Simple Summary:**

Current methods for the detection of brain malignancies often display low sensitivity and specificity. Noninvasive biomarkers can complement imaging techniques to improve the diagnosis of these tumors. The aim of this study was to identify circulating miRNAs in serum exosomes useful in all phases of the diagnostic and therapeutic path of patients with malignant brain lesions. Our data show a signature of exosomal miRNAs useful for the differential diagnosis of brain metastases and for monitoring tumor evolution over time.

**Abstract:**

Circulating miRNAs are increasingly studied and proposed as tumor markers with the aim of investigating their role in monitoring the response to therapy as well as the natural evolution of primary or secondary brain tumors. This study aimed to evaluate the modulation of the expression of three miRNAs, miR-21, miR-222 and miR-124-3p, in the serum exosomes of patients with high-grade gliomas (HGGs) and brain metastases (BMs) to verify their usefulness in the differential diagnosis of brain masses; then, it focused on their variations following the surgical and/or radiosurgical treatment of the BMs. A total of 105 patients with BMs from primary lung or breast cancer, or melanoma underwent neurosurgery or radiosurgery treatment, and 91 patients with HGGs were enrolled, along with 30 healthy controls. A significant increase in miR-21 expression in serum exosomes was observed in both HGGs and BMs compared with healthy controls; on the other hand, miR-124-3p was significantly decreased in BMs, and it was increased in HGGs. After the surgical or radiosurgical treatment of patients with BMs, a significant reduction in miR-21 was noted with both types of treatments. This study identified a signature of exosomal miRNAs that could be useful as a noninvasive complementary analysis both in the differential diagnosis of BMs from glial tumors and in providing information on tumor evolution over time.

## 1. Introduction

Brain metastases (BMs) represent important causes of morbidity and mortality in cancer patients and are the most common intracranial tumors, occurring in approximately 10–30% of adult patients with cancer [1]. The risk of developing BMs varies according to the primary-tumor type. The most frequent primary tumors that lead to the development of cerebral metastases are lung cancer, breast cancer and melanoma, accounting for 67–80% of all BMs [2]. The incidence of these metastases has increased in recent years for the availability of improved imaging techniques, which aid early diagnosis. Approximately 40–50% of patients with metastatic brain cancer have a single metastasis, while multiple lesions are diagnosed in approximately 50–60% [3] and are associated with poor prognosis.

Survival seems to be primarily determined by the activity of extracerebral disease and systemic treatment options. The treatment of BMs continues to play a major role in preventing disease progression and in the deterioration of the neurological status and of the quality of life to limit the morbidity associated with systemic therapies [4].

Treatment options for BMs include surgical resection with or without radiotherapy of the whole brain (WBRT), an independent method of WBRT, radiosurgery (SRS), SRS + WBRT and chemotherapy. Stereotactic radiosurgery is most frequently used in the treatment of solitary or multiple BMs, provided that the overall volume is less than 20 cc. The treatment’s effects on solitary BMs are comparable to those of surgical treatment [5]. This method is based on the strict immobilization of the patient, the precise determination of the boundaries of the lesion and the use of a computer system for treatment planning and for the delivery of a high dose of radiation in a well-defined area of the tumor, sparing healthy tissue as much as possible. Treatment planning is based on the fusion of a series of Computed Tomography (CT) images and Magnetic Resonance Imaging (MRI) to precisely determine the volume of the target [6].

When a brain mass is detected in patients where extracranial malignancies are not diagnosed, conditions such as primary brain tumors, abscesses or granulomas are usually taken into consideration in differential diagnosis [7,8]. MRI is very sensitive but not specific in identifying these lesions, and in the last 5 years, the clinical management of these patients has changed based on molecular biology for differential diagnosis, the monitoring of disease evolution and response to therapies.

Recently, there is an increasing interest in liquid biopsy in the field of neuro-oncology. The search for a non-invasive test that can give information on the state of the disease, in primary or secondary brain tumors, is the challenge of recent years. Among these, the role of circulating biomarkers in the cerebrospinal fluid, such as circulating tumor DNA, microRNAs and metabolites [9], and blood biomarkers, the latter being more accessible for easy sampling [10], emerged in the studies.

Exosomes, small extracellular vesicles detectable in the blood stream, are emerging as promising sources of diagnostic and prognostic biomarkers in many forms of cancer, as they contain different types of molecules selectively derived from the cell of origin, including small non-coding RNAs, the microRNAs (miRNAs) [11]. Many of these miRNAs regulate key intrinsic tumor processes such as proliferation, apoptosis, migration and progression [12]. Exosomes can be isolated from peripheral blood and miRNAs can be measured by widely used techniques. It has been shown that it is possible to discriminate the exosomes secreted by the tumor that contain the microRNAs and peptides necessary for its growth and survival [10]. The altered expression levels of some miRNAs, which has been observed in brain tumor tissue, has been also studied as a biomarker to predict survival and response to therapy [13,14].

We previously showed that the expression of three miRNAs—miR-21, -222 and -124-3p—in serum exosomes from patients with high-malignancy-grade gliomas (HGGs) was significantly increased compared with patients with low-malignancy-grade gliomas (LGGs) or healthy subjects and it drastically decreased after the surgical removal of the tumor [15].

The aim of this study was to demonstrate how circulating miRNAs in serum exosomes can be useful in all phases of the diagnostic–therapeutic path of patients with malignant brain lesions.

To ascertain the potential role in the differential diagnosis of malignant brain masses, we compared the expression of circulating miR-21, -222 and -124-3p in patients with different primary and secondary lesions and in healthy controls. In order to evaluate whether these miRNAs can provide information about the response to treatment, we analyzed their expression levels in the immediate post-operative period and one month after GK treatment. 

We found that the reduced expression of miR-124-3p associated with increased levels of miR-21 in serum exosomes was useful for the differential diagnosis of BMs. Mir-21 expression was significantly reduced both after the surgical removal of the tumor and GK treatment. To the best of our knowledge, this is the first study that compares the different expression of three miRNAs in patients undergoing surgery and radiosurgery. 

## 2. Results

### 2.1. Increased Levels of miR-21 Associated with Decreased miR-124-3p in Serum Exosomes Characterized Patients with BMs

We previously demonstrated that the pre-operative miR-21, miR-222 and miR-124-3p expression in exosomes derived from the serum of patients with HGGs was significantly higher than that in healthy controls [15]. Moreover, in a small number of patients with secondary BMs, we found that miR-21 expression, but not miR-222 or miR-124-3p expression, was also increased compared with healthy controls, thus suggesting that these miRNAs could help the differential diagnosis between HGGs and non-glial BMs. Therefore, we extended the analysis to a larger group of patients with different primary tumors in comparison with patients with HGGs and healthy controls, as listed in Table 1.

**Figure 1:**
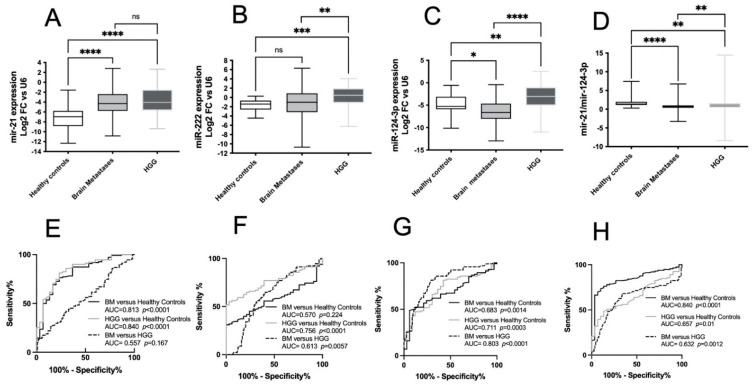
*miRNA expression in serum exosomes of healthy controls and patients with BMs and HGGs*. miR-21 (**A**), -222 (**B**) and -124-3p (**C**) expression in serum exosomes was measured with real-time qPCR with TaqMan probes and normalized to U6 snRNA. Data are expressed as Log2FC. Ratio miR-21/miR-124-3p (**D**). Lines in the boxes are median, and 25% and 75% percentile values; bars indicate minimum and maximum values. Comparisons between healthy controls and patients with BMs and HGGs were analyzed with non-parametric Kruskal–Wallis tests. Differences were considered statistically significant with *p* < 0.05 (*), *p* < 0.01 (**), *p* < 0.001 (***) and *p* < 0.0001 (****). Median values and CIs are indicated in Table 2. (**E**–**H**) ROC curves showing sensitivity and specificity for miR-21 (**E**), -222 (**F**), -124-3p (**G**) expression and ratio miR-21/miT-124-3p (**H**). AUC is area under the curve. ns means “not significant”.

The data reported in Figure 1 confirm that the expression of miR-21 in serum exosomes from patients with BMs or HGGs was significantly higher than that in healthy controls (Figure 1A) (*p* < 0.0001). No differences were found in the miR-21 expression between patients with BMs and HGGs. The miR-222 expression in patients with BMs was not significantly different from that in healthy controls, whereas it was significantly lower than that in patients with HGGs, in agreement with our previous data [15] (Figure 1B). Very interestingly, the expression of miR-124-3p was significantly lower than that in both healthy controls and patients with HGGs (Figure 1C). We also calculated the ratio between miR-21 and miR-124-3p expression and found that it was very significantly decreased in patients with BMs, with a median value of 0.67 compared with the 1.36 value for healthy controls or the 1.07 value for patients with HGGs (Figure 1D). To evaluate the diagnostic performance and discriminatory accuracy of these miRNAs, ROC curves were constructed. miR-21 (Figure 1E) was robust for discriminating both BMs and HGGs from healthy controls, with areas under the curve (AUCs) of 0.813 (95% CI: 0.726–0.900) and 0.840 (95% CI: 0.758–0.921), respectively, while it was not able to differentiate BMs from HGGs, with an AUC of 0.557 (95% CI: 0.476–0.637). As shown in Figure 1F, miR-222 was a good biomarker only for HGGs vs. healthy controls with an AUC of 0.756 (95% CI: 0.674–0.837), in agreement with our previous data [15]. MiR-124-3p appeared to be the best biomarker for distinguishing patients with BMs from those with HGGs (Figure 1G) with the highest AUC of 0.803 (95% CI: 0.742–0.864). The ROC curves of the miR21/miR-124-3p ratio indicated that this ratio did not improve the diagnostic accuracy of miR-21 alone for the differential diagnosis of BMs from HGGs (Figure 1H). 

To help the differential diagnosis of BMs and HGGs from healthy controls and that of BMs from HGG, cutoff thresholds were calculated as reported in Table 3. Considering together sensitivity and specificity, miR-21 was a good indicator to confirm in neuroradiological images the presence of a brain tumor, and miR-124-3p was useful to indicate a metastatic brain cancer. These results suggest that the reduction in the expression of miR-124-3p associated with the increased levels of miR-21 in serum exosomes could become a useful biomarker pattern to discriminate against non-glial malignant brain masses in the presence of a small-size neuroimage previous to histological characterization.

### 2.2. The Lowest Median miR-124-3p Expression Was Found in the Group of Patients with BMs from Melanoma

To evaluate whether miR-21 and miR-124-3p were differentially expressed in patients with BMs from different neoplasias, we selected the three most numerous groups of patients representing lung cancer, breast cancer and melanoma, as listed in Table 4, and compared each group to the controls.

**Figure 2:**
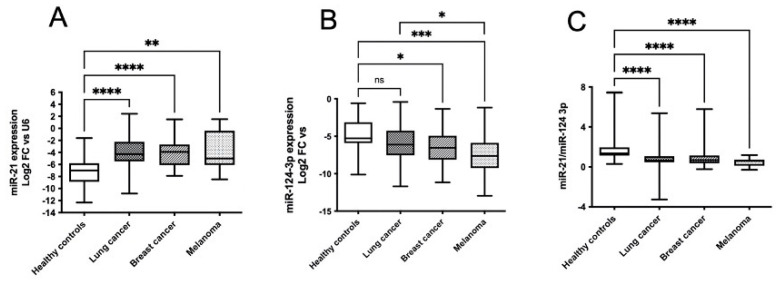
*miRNA expression in serum exosomes of healthy controls and patients with BMs from lung cancer, breast cancer and melanoma*. miR-21 (**A**) and -124-3p (**B**) expression in serum exosomes was measured as indicated in Figure 1. Ratio miR-21/miR-124-3p (**C**). Comparisons between healthy controls and patients with BMs were analyzed with non-parametric Kruskal–Wallis tests. Differences were considered statistically significant with *p* < 0.05 (*), *p* < 0.01 (**), *p* < 0.001 (***) and *p* < 0.0001 (****). ns means “not significant”.

As shown in Figure 2A, miR-21 was significantly increased in all groups of patients compared with healthy controls. No different miR-21 expression among the three groups of patients was observed. Median miR-124-3p expression in patients with BMs from lung cancer was lower than that in healthy controls, although this difference did not reach statistical significance (Figure 2B). In patients with BMs from both breast cancer and melanoma, the median expression values of miR-124-3p were −6.54 and −7.64, respectively, significantly lower than those for controls (−5.28). Very interestingly, the lowest median miR-124-3p expression was found in the group of patients with BMs from melanoma (Table 4 and Table 5). Moreover, the median expression in this group was significantly lower than that in patients with BMs from lung cancer. The ratio between mir-21 and mir-124-3p expression was significantly lower in the three groups of patients compared with the healthy controls, whereas no differences were observed among these groups of patients (Figure 2C).

### 2.3. miR-21 Expression Was Reduced after Both Surgery and Gamma-Knife Treatment of BMs

**Figure 3:**
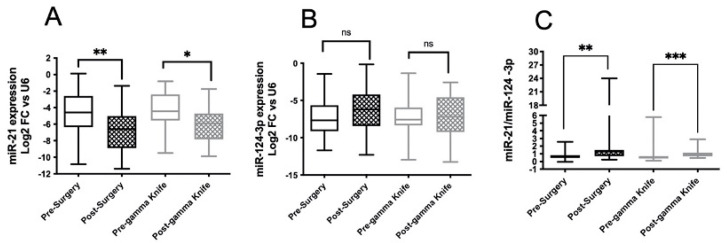
*miRNA expression in serum exosomes of patients with BMs before and after surgery or GK*. miR-21 (**A**) and -124-3p (**B**) expression in serum exosomes was measured in blood samples collected both before and one week after surgery or one month after GK, as described in Figure 1. Ratio miR-21/miR-124-3p (**C**). Comparisons between pre- and post-surgery, and post-GK were analyzed with Wilcoxon matched-pair signed-rank tests. Differences were considered statistically significant with *p* < 0.05 (*), *p* < 0.01 (**), *p* < 0.001 (***). Median values and CIs are indicated in Table 6. ns means “not significant”.

We previously described a marked reduction in exosomal miR-21, miR-222 and miR-124-3p expression in patients with HGGs upon the surgical removal of the tumor [15]. Moreover, we recently found that the increased expression of these miRNAs at the post-operative follow-up was associated with the progression of HGGs [16]. In order to verify whether miR-21 and miR-124-3p expression can be useful to assess the effect of treatment in patients with secondary BMs, we studied the miRNA expression in serum collected both before and after neurosurgery or GK. We separately analyzed patients undergoing a surgical removal of BMs and patients treated with GK. Blood samples were collected one week or one month after neurosurgery or GK, respectively. As shown in Figure 3A, miR-21 expression was significantly reduced after the surgical removal of the tumor, according to the obtained data of patients with HGGs [15]. Interestingly, similar results were also observed after GK treatment. These findings indicate that the origin of the dysregulated expression of miRNAs in the serum exosomes of the patients included in this study was bona fide prevalently derived from the tumor mass localized in the brain. There were no differences between pre- and post-surgery or pre- and post-GK in miR-124-3p expression (Figure 3B), whereas a change in the ratio between miR-21 and miR-124-3p expression was found in both groups of patients (Figure 3C). In order to ascertain that the reduction in miR-21 expression was due to the ablation of the tumor mass localized in the brain by neurosurgery or GK treatment, we selected patients in whom the primary tumor had been surgically excised and no other metastases besides brain ones were detectable.

**Figure 4:**
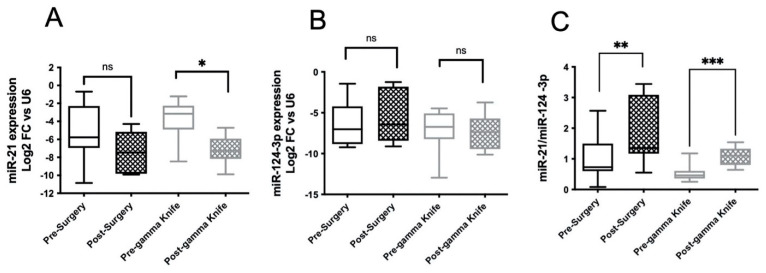
miRNA expression in serum exosomes of patients with BMs before and after surgery or GK with primary tumor surgically excised and no other metastases except brain ones. miR-21 (**A**) and -124-3p (**B**) expression in serum exosomes was measured in blood samples collected both before and one week after surgery or one month after GK, as described in Figure 1. Ratio miR-21/miR-124-3p (**C**). Comparisons between pre- and post-surgery, and post-GK were analyzed with Wilcoxon matched-pair signed-rank tests. Differences were considered statistically significant with *p* < 0.05 (*), *p* < 0.01 (**), *p* < 0.001 (***). ns means “not significant”.

The data reported in Figure 4A show a marked reduction in miR-21 expression after both neurosurgery and GK, with median values of −5.78 vs. −7.46 and −3.15 vs. −7.27 in patients who underwent surgery and GK, respectively (Table 5). In addition, in these selected cases, no differences were observed in miR-124-3p expression (Figure 4B), whereas in agreement with the data reported in Figure 3C, the ratio between miR-21 and miR-124-3p expression was significantly changed in both groups of patients (Figure 4C). These findings further suggest that BMs contributed to the dysregulated expression of miR-21 in the serum exosomes of these patients.

## 3. Discussion

BMs represent a new challenge in the clinical management of cancer patients, as they can be detected at the same time as the primary tumor or can be the initial sign of a still unknown primary malignancy. 

The differential diagnosis between HGGs and BMs in the case of a single brain lesion with no known primary tumors remains one of the challenges for neuroradiology, although recently, advanced neuroimaging studies have been providing new evaluation parameters [17]. Thus, the aim of our study was to ascertain whether the detection of biomarkers in bodily fluids could help anticipating the nature of the brain mass with a limited invasive approach and to collect information on the efficacy of different treatment approaches to remove BMs.

The main finding of the present study is that the increased expression of miR-21 and decreased miR-124-3p in serum exosomes provided a molecular signature characterizing BMs in the presence of a small-size neuroimage. In agreement with our previously published data [15], the exosomal miR-21 expression in patients with both primary and secondary brain tumors was higher than that observed in healthy controls. As shown by our data, this miRNA is a very sensitive and specific biomarker for differentiating brain tumors from healthy controls in the presence of a brain mass of unknown origin. Up-regulated miR-21 expression in different types of cancer, including brain tumors, has been extensively described [18]. Its role in the carcinogenesis process, progression, invasion and migration has been widely ascertained. Our findings, showing increased miR-21 expression in the serum exosomes of BM patients, are consistent with the higher miR-21 expression described both in the brain tissue and in the cerebrospinal fluid of patients with BMs from lung cancer [19,20]. Notably, in our study, not only patients with BMs from lung cancer but also patients with BMs from other neoplasias were characterized by up-regulated exosomal miR-21 expression.

In the presence of brain masses at neuroimaging assessment with inconclusive diagnosis, exosomal miR-21 did not aid the differentiation between primary and secondary tumors. In this respect, the combined assessment of miR-21 and miR-124-3p identified patients with BMs, as miR-124-3p was down-regulated in BMs, whereas it was up-regulated in HGG patients. Cutoff values obtained from our data indicated that miR-124-3p expression in serum exosomes was both a sensitive and a specific marker for the differential diagnosis between BMs and HGGs. Due to the small number of patients with BMs from melanoma, reliable cutoff values for patients with different primary tumors could not be obtained. However, it was interesting to note that the lowest median miR-124-3p expression was observed in BMs from melanoma. Thus, when BMs are the initial presentation of a still unknown neoplasia, very low miR-124-3p expression combined with high miR-21 expression can provide important hints to correctly screen for the presence of an undiagnosed melanoma. MiR-124 is one of the most highly expressed miRNAs in the brain, affecting important biological functions [21]. The PIM1 protein, involved in cell proliferation, cell cycle and apoptosis in various tumors has been demonstrated to be one of the targets of miR-124 [22]. Although miR-124 is downregulated in many different types of cancers, it has been reported to be increased in cancers of glial origin such as glioblastomas and astrocytomas [23]. This suggests that the increased exosomal miR-124-3p expression found here and previously reported [15] in the serum of patients with HGGs derives from highly expressed miR-124 in the brain tumor of these patients. On the other hand, the low exosomal mir-124-3p expression observed in the serum of patients with BMs could reflect the down-regulation of this miRNA described in cancers other than gliomas. Margolyn-Miller et al. proposed a diagnostic stratification of ependymomas (also tumors of glial origin) based on the expression of mir-124-3p, as they were shown to be independent prognostic factors for PFS [24].

The other novel key finding of this study is the significant decrease in exosomal miR-21 expression and the increase in the miR21/miR124-3p ratio both after the surgical removal and GK treatment of BMs. Therefore, circulating miR-21 expression could become a marker to assess the therapeutic efficacy of the treatment of BMs. As a matter of fact, MRI performed after surgery or GK in these patients showed the effectiveness of the treatment.

Our results are consistent with data reporting decreased levels of miR-21 after WBRT in patients with BMs from lung cancer [25]. Interestingly, a meta-analysis proposes miR-21 as a biomarker to predict and monitor response to radiotherapy in cancer [26]. 

Differently from miR-21, we here showed no significant changes in miR-124-3p expression after surgery or GK treatment, further suggesting that the levels of exosomal miR-124-3p in serum were due to the primary tumor of the patients with BMs, whilst the tumor mass localized in the brain contributed to the upregulated miR-21 expression in the serum exosomes of these patients.

There is always a greater interest in the study of miRNAs as tumor biomarkers and in their role in prognosis and response to therapy. Interestingly, miRNA changes following ionizing radiations have been reported [27,28], thus emphasizing their role in the radiosensitivity and radiation resistance to therapy.

Other studies have demonstrated the role of miRNAs in response to radiations. For instance, by blocking the action of miR-21 through anti-miR, cells were more radiosensitive to the radiation emanating from a special cellular irradiator [29]. Another report showed variations in miR-34 in response to bleomycin and gamma-radiation treatment in specific cell lines involving the P53 pathway [30].

Chakraborty et al. demonstrated the role of radiation in miRNAs and circulating metabolites by irradiating mice with a lethal dose in order to study the activation networks of tissue damage from gamma radiation with Cobalt-60 [31].

To the best of our knowledge, this is the first report showing dynamic changes in exosomal miR-21 expression after GK treatment of BMs in vivo.

As shown in Figure 3, the serum levels of miR-21 underwent a significant reduction both after surgery and after GK treatment. The miR-21/ miR124 ratio also showed a significant variation.

This takes on a greater significance if, by removing the bias of other disease localizations, we consider patients who only have BMs, in the absence of primitive unoperated or other body metastases (Figure 4). These preliminary results lay the foundation for a longer follow-up over time in which to study the evolution of the disease and the possible prognostic role of miRNAs in detecting any recurrences of the disease in advance.

Apart from the role in differentiating BMs from glial tumors, the miRNA signature here identified could provide information on tumor evolution over time. The collection of blood samples together with clinical evaluation during longitudinal follow-ups of a larger number of patients with BMs would be needed to ascertain whether increased levels of miR-21 in serum exosomes indicate tumor progression.

## 4. Materials and Methods

### 4.1. Clinical Features

In total, 105 patients aged between 18 and 75 with first clinical and/or histological diagnosis of BMs from primary lung, breast and kidney cancer and melanoma, undergoing treatment with GK or surgery and 91 patients with HGGs were enrolled after signing their informed consent. In total, 30 healthy controls with no history of cancer or abnormalities in laboratory tests were also enrolled. The local Ethics Committee approved this study, which was performed in accordance with the Declaration of Helsinki (MiR2015; CESC_VR-RO #44535, 29/09/2015) and Good Clinical Practice guidelines. The approval for the publication of these data was obtained from Ethics Committee and from all the patients enrolled in the study via written informed consent. Demographic and clinical features are reported in Table 1.

### 4.2. Study Design

Blood samples were collected before surgery or GK. In some patients with BMs, one week after surgery (23 patients) or one month after GK (23 patients) a second blood sample was obtained. After clotting, samples were centrifuged and stored at −80 °C.

### 4.3. Analysis of miRNA Expression

miR-21, miR-222 and miR-124-3p expression in serum exosomes was measured as described [15]. Briefly, exosomes were isolated from 0.5 mL of serum using ExoQuick (System Biosciences-Euroclone SpA, Pero, Milan, Italy) precipitation solution. RNA was isolated from exosomes using an exoRNeasy Serum/Plasma kit (Qiagen s.r.l., Milan, Italy) and miRNA expression levels were quantified with RT-qPCR with specific TaqMan probes. U6snRNA was used as a normalizer.

### 4.4. Radiosurgical Techniques

GK SRS was performed in 65 patients with BMs—mean age of 65.14 years (range: 43–83), 23 M and 42 F—with 106 lesions. A blood sample was obtained from each patient before treatment and a second sample 1 month after GK SRS.

Technical details of the GK procedure have already been fully described in the literature [6,32,33].

Briefly, the procedure consisted of positioning a Leksell stereotactic frame (Elekta Instrument SA, Stockholm, Sweden) on the patient’s head under local anesthesia.

Neuroradiological localization was routinely performed using stereotactic MRI with specific algorithms and sequences (1 mm isovoxel volumetric with double-dose gadolinium-enhanced images). SRS procedures were performed with model C 201-source Co60 Leksell Gamma Unit and, since June 2008, with Gamma Knife (GK) Perfexion (both obtained from Elekta Instruments). Three-dimensional treatment planning was developed using Leksell Gamma Plan (versions 4.12, 5.34, 8.3 and 10.1.1; Elekta Instruments). Mean and range dose-planning parameters were as follows: gross target volume (GTV 2.76 cc, 0.01–20.4 cc), prescription dose (PD 19.97 Gy, 12.0–23.0 Gy), prescription isodose (PI 50%, in all cases), maximum dose (MD 39.94 Gy, 24.0–46.0 Gy) and shot number (5.81, 1–25). SRS was characterized by PD and MD intensity delivered in compliance with the brain stem, the optic nerve, the chiasm and the pituitary peduncle.

### 4.5. Surgical Techniques

Regarding the clinical and neuroimaging characteristics, 40 patients with BMs underwent neurosurgery by craniotomy, with the aid of an intraoperative microscope. Of these, 24 were female, and 16 were male, with an average age of 64 years.

### 4.6. Statistics

All the statistics were carried out with GraphPad Prism 9.0 as specified in the figure legends. Differences were considered statistically significant with *p* < 0.05 (*), *p* < 0.01 (**), *p* < 0.001 (***) and *p* < 0.0001 (****). ROC curves were plotted using GraphPad Prism 9.0 software. Cut-off values were calculated by optimizing the sums of sensitivity and specificity (Youden’s Method) for each miRNA and for the miR-21/miR124-3p ratio.

## 5. Conclusions

The differential diagnosis of brain tumors remains one of the challenges for neuroradiology. Our study demonstrates that the increased expression of miR-21 and decreased miR-124-3p in serum exosomes discriminated BMs from HGGs. Decreased exosomal miR-21 expression after both the surgical removal and GK treatment of BMs indicated that this miRNA could complement neuroradiological imaging in the longitudinal follow-up of patients with BMs. To the best of our knowledge, this is the first report showing dynamic changes in exosomal miR-21 expression after the treatment of BMs with GK.

## Figures and Tables

**Table 1:** Demographic and clinical features of healthy controls and patients with brain metastases (BMs) and high-grade gliomas (HGGs).

Category	n	Age (Years) Mean ± SEM	Males (n)	Females (n)
Healthy Controls	30	41 ± 12	13	17
BMs	105	64 ± 11	38	67
HGGs	91	56 ± 14	60	31

**Table 2:** Median values and 95% CIs of miR-21, miR-222, miR-124-3p and ratio miR-21/miR-124-3p in healthy controls and patients with brain metastases (BMs) and high-grade gliomas (HGGs).

	BMs	HGGs	Healthy Controls
*miR-21*	−4.3 (95% CI: −4.78–−3.81)	−4.06 (95% CI: -4.78–−3.81	−6.9 (95% CI: −8.04–−6.09)
*miR-222*	−1.03 (95% CI: −1.62–−0.51)	−0.48 (95% CI: −0.38–0.94)	−1.42 (95% CI: −2.16–−0.42)
*miR-124-3p*	−6.63 (95% CI: −7.22–−5.77)	−3.06 (95% CI: −4.11–−2.32)	−5.28 (95% CI: −5.67–−4.41)
*miR-21/miR-124-3p*	0.67 (95% CI: 0.58–0.71)	1.07 (95% CI: 0.89–1.32)	1.36 (95% CI: 1.24–1.63)

**Table 3:** Cutoff values for exosomal miRNA expression.

	miRNA	Cutoff	Youden Index	Sensitivity	Specificity
***BMs vs. healthy controls***					
	miR-21	−5.86	0.530	0.764	0.767
	miR-222	0.42	0.310	0.310	1.000
	miR-124-3p	−6.86	0.426	0.487	0.939
	miR-21/ miR-124-3p	0.96	0.636	0.736	0.900
***HGGs vs. healthy controls***					
	miR-21	−5.76	0.580	0.813	0.767
	miR-222	0.10	0.519	0.549	0.970
	miR-124-3p	−5.06	0.389	0.813	0.576
	miR-21/ miR-124-3p	1.05	0.361	0.495	0.867
***BMs vs. HGGs***					
	miR-21	−3.18	0.125	0.407	0.718
	miR-222	0.06	0.242	0.560	0.681
	miR-124-3p	−5.06	0.521	0.813	0.708
	miR-21/ miR-124-3p	0.80	0.336	0.681	0.655

Cutoff values were calculated by optimizing the sums of sensitivity and specificity (Youden’s Index) for each miRNA.

**Table 4:** Demographic and clinical features of patients with brain metastases (BMs).

Category	n	Age (Years) Mean ± SEM	Males (n)	Females (n)
Secondary brain metastases from pulmonary cancer	35	64 ± 11	19	16
Secondary brain metastases from breast cancer	33	64 ± 12	0	33
Secondary brain metastases from melanoma	13	63 ± 12	8	5
Other tumors	24	64 ± 11	11	13

**Table 5:** Median values and 95% CIs of miR-124-3p in healthy controls and patients with brain metastases (BMs) from lung cancer, breast cancer and melanoma.

	Healthy Controls	Lung Cancer	Breast Cancer	Melanoma
*miR-124-3p*	−5.28 (95% CI: −5.67–−4.41)	−6.125 (95% CI: −7.24–−4.90)	−6.54 (95% CI: −7.82–−5.23)	−7.64 (95% CI: −9.41–−5.26)

**Table 6:** Median values and 95% CIs of miR-21 in patients with brain metastases (BMs) pre- and post-surgery and pre- and post-gamma knife.

	Pre-Surgery	Post-Surgery	Pre-Gamma Knife	Post-Gamma Knife
*miR-21*	−5.78 (95% CI: −10.85–−0. 69)	−7.46 (95% CI: −9.92–−4.30)	−3.15 (95% CI: −5.55–−2.22)	−7.27 (95% CI: −9.61–−5.76)

## Data Availability

The approval for the publication of these data was obtained from Ethics Committee and from all the patients enrolled in the study via written informed consent.

## Abbreviations

BM	brain metastasis
WBRT	radiotherapy of the whole brain
SRS	radiosurgery
CT	Computed Tomography
MRI	Magnetic Resonance Imaging
miRNAs	microRNAs
HGG	high malignancy grade gliomas
LGG	low malignancy grade gliomas
GK	gamma knife
